# Molecular characterization of novel mosquito-borne *Rickettsia* spp. from mosquitoes collected at the Demilitarized Zone of the Republic of Korea

**DOI:** 10.1371/journal.pone.0188327

**Published:** 2017-11-20

**Authors:** Alice N. Maina, Terry A. Klein, Heung-Chul Kim, Sung-Tae Chong, Yu Yang, Kristin Mullins, Ju Jiang, Heidi St. John, Richard G. Jarman, Jun Hang, Allen L. Richards

**Affiliations:** 1 Viral and Rickettsial Diseases Department, Naval Medical Research Center, Silver Spring, Maryland, United States of America; 2 65^th^ Medical Brigade, Medical Department Activity-Korea, Unit 15281, Seoul, South Korea; 3 65^th^ Medical Brigade, Medical Department Activity-Korea, Unit 15247, Seoul, South Korea; 4 Viral Diseases Branch, Walter Reed Army Institute of Research, Silver Spring, Maryland, United States of America; 5 University of Maryland, School of Medicine, Department of Pathology, Baltimore, Maryland, United States of America; Swedish University of Agricultural Sciences, SWEDEN

## Abstract

Rickettsiae are associated with a diverse range of invertebrate hosts. Of these, mosquitoes could emerge as one of the most important vectors because of their ability to transmit significant numbers of pathogens and parasites throughout the world. Recent studies have implicated *Anopheles gambiae* as a potential vector of *Rickettsia felis*. Herein we report that a metagenome sequencing study identified rickettsial sequence reads in culicine mosquitoes from the Republic of Korea. The detected rickettsiae were characterized by a genus-specific quantitative real-time PCR assay and sequencing of *rrs*, *gltA*, 17kDa, *ompB*, and *sca4* genes. Three novel rickettsial genotypes were detected (*Rickettsia* sp. A12.2646, *Rickettsia* sp. A12.2638 and *Rickettsia* sp. A12.3271), from *Mansonia uniformis*, *Culex pipiens*, and *Aedes esoensis*, respectively. The results underscore the need to determine the *Rickettsia* species diversity associated with mosquitoes, their evolution, distribution and pathogenic potential.

## Introduction

The genus *Rickettsia* contains a diverse collection of obligate intracellular bacteria that are primarily arthropod-associated [1]. In the last few decades, new species and strains of *Rickettsia* and geographic spread of known rickettsiae once thought to be geographically restricted have been discovered at an increasing rate from a varied host range [2–5].

In the Republic of Korea (ROK), several tick-borne spotted fever group rickettsiae (SFGR) have been detected by molecular methods including *R*. *japonica* detected in *Haemaphysalis longicornis* collected from Chungju, North Chungcheong province by flagging [6]; *Rickettsia monacensis* in *Ixodes nipponensis* collected from small mammals, South Jeolla, Gyeonggi and Gangwon provinces [7, 8]; and several unidentified *Rickettsia* spp. detected in *Haemaphysalis* and *Ixodes* spp. collected by tick drag from five provinces [9], and in northern Gyeonggi and Southwestern provinces [10, 11]. *Rickettsia japonica* was isolated from a patient and confirmed by PCR and sequencing [12]. Additionally, antibodies reactive to SFGR were detected in sera from febrile Korean patients [13, 14] and from US Army soldiers that were deployed to the ROK that conducted training at US/ROK operated training sites located near the demilitarized zone (DMZ) [15]. In addition to tick-borne rickettsiae, flea-borne rickettsiae, *R*. *felis* and *R*. *typhi*, were identified in fleas collected from live-captured small mammals from Gyeonggi province [16].

Historically, the vector-hosts for rickettsiae have been ticks, fleas, lice and mites [5], but recent studies have also shown the existence of rickettsiae in other invertebrates including leeches, ladybirds, sheep keds, and mosquitoes [1, 3, 17, 18]. A recent study also demonstrated that *Anopheles gambiae* may act as a potential vector for *R*. *felis* through induction of transient rickettsemia in mice [19]. This study and the observations of the presence of *R*. *felis* infections in humans, particularly in regions that are endemic for malaria, have sparked a lot of interest in mosquitoes as possible vectors for *R*. *felis* [4, 5, 20, 21].

Other evidence that suggests an association between rickettsiae and mosquitoes includes an early study that demonstrated the presence of an intracellular *Rickettsia*-like microorganism in the gonad cells of *Culex pipiens* in 1924 [22], which was later identified and named *Wolbachia pipientis* [23]. With the development of mosquito cell lines (*Aedes albopictus* Aa23, *Ae*. *albopictus* C6/36, and *An*. *gambiae* Sua5B), it has been determined that *Wolbachia* and *Rickettsia* species can utilize gonad cells for propagation [24]. These results have led several investigators to search for the presence of rickettsiae in mosquitoes. Several studies have now detected *Rickettsia* species by molecular techniques in mosquitoes from China [18, 25, 26] and Africa (*Aedes* spp. [21, 27], *Anopheles* spp. [21, 23], and *Mansonia uniformis* [21]). We report on the utilization of next-generation sequencing (NGS) technology using metagenome sequencing based approach to detect rickettsiae in field-collected mosquitoes near/in the DMZ of the ROK. Subsequently, additional gene sequencing was performed to further characterize the *Rickettsia* agents detected by NGS.

## Materials and methods

**Fig 1:**
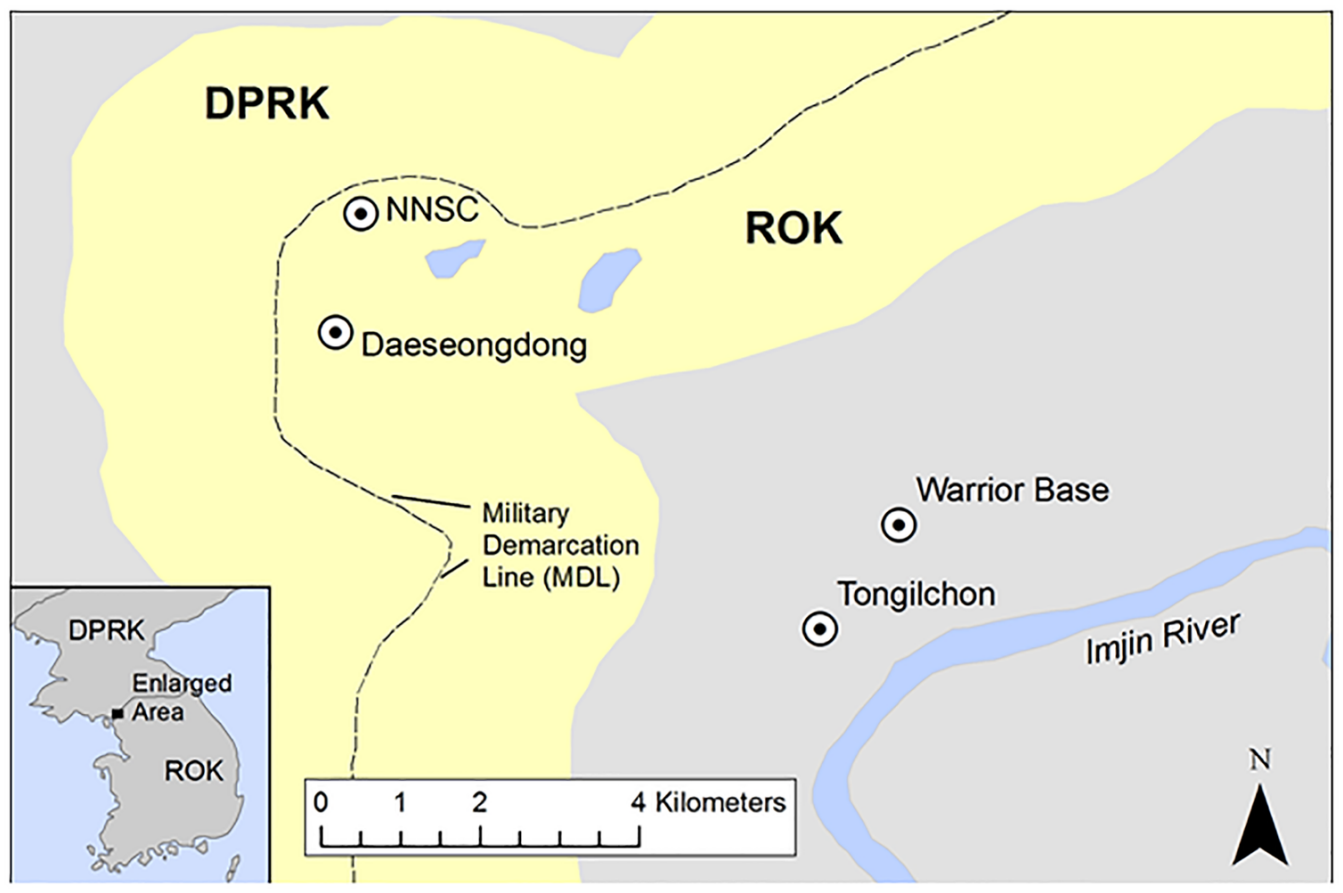
Map of the northern part of Gyeonggi province denoting collection sites of mosquitoes at NNSC (Neutral Nations Supervisory Commission camp adjacent to the Panmunjeom), Daeseongdong (located inside the Demilitarized Zone), Warrior Base (US Army training site) and Tongilchon (beef farm) located 2 km and 3 km, respectively from the MDL southern boundary of the Demilitarized Zone.

Mosquitoes were collected by Mosquito Magnet^®^ (Pro model, Woodstream Corp. Lititz, Pennsylvania, USA) at military installations and training sites and villages near/in the DMZ [Collection sites: the Neutral Nations Supervisory Commission (NNSC) camp (37°57'16.39" N,26°40'50.03" E), Daeseongdong village (37°56'26.92" N, 126°40'37.42" E), Warrior Base Training Area (WBTA) (37°55'17.01" N, 126°44'30.22" E), and Tongilchon (beef farm ≈50 cattle) (37°54'32.18" N, 126°44'01.88" E)] in northern Gyeonggi Province, ROK from May-October 2012 (Fig 1). The NNSC camp is located inside the DMZ and is adjacent to the Military Demarcation Line (MDL) and Panmunjeom. Daeseongdong village is located inside the DMZ and adjacent to the MDL with approximately 200 residents. WBTA (a US Army training site) and Tongilchon village, with ≈200 residents, are located approximately 3 km from the southern entrance to the DMZ.

Mosquitoes were identified morphologically using standard keys [28, 29] and then placed in pools of up to 30 mosquitoes in 1.5 ml cryovials by species, date of collection, and collection site. The pooled specimens were homogenized in 0.6 ml of cell culture medium by bead-beating for 1 min using BioSpec Mini-BeadBeater-16 (Bio Spec Products Inc., Bartlesville, OK). After centrifugation at 6,000 × g for 10 min at 4°C, 130 μl clear supernatant was removed and treated with RNase free-DNase (QIAGEN Sciences, Germantown, MD) and subjected to nucleic acid extraction using QIAamp viral RNA kit (QIAGEN Sciences, Germantown, MD).

Purified nucleic acid samples were used in random reverse transcription and PCR amplification as described previously [30]. Nuclease-Free water (Invitrogen) (used as a blank control) and viral RNA of MS2 bacteriophage, a positive-sense single-stranded RNA virus (Roche Applied Science, Indianapolis, IN) were processed in parallel in separate reaction wells and analyzed to monitor the level of cross contamination. Random amplicons were sequenced using MiSeq sequencing system and reagents (Illumina, San Diego, CA). Sequence reads were subjected to metagenomics analyses using a bioinformatics pipeline [31], for sequence-based taxonomical identification of components in the specimens.

For mosquito specimens with rickettsial sequence reads identified in random metagenome sequencing, a total of 200 μl of the remaining cell culture medium homogenates were subjected to DNA extraction using QIAamp blood and tissue kits (QIAGEN), and the purified DNA was eluted in 100 μl of elution buffer. Two negative extraction controls (ECs) using molecular biology grade water (Invitrogen) were incorporated to ensure that the process was free from sample-to-sample contamination. DNA was diluted later using 10-fold serial dilutions (10–1000×) to reduce the effect of PCR inhibitors. The DNA preps were tested by a genus-specific quantitative real-time PCR (qPCR) assay (Rick17b) that amplifies and detects a 115-bp segment of the 17-kDa antigen gene [32].

PCR and nested PCR (nPCR) amplification and sequencing of *rrs*, *gltA*, 17 kDa antigen gene, *ompB*, *ompA*, and *sca4* gene fragments were attempted on all Rick17b positive DNA preparations using primers and procedures previously described [33–36] and summarized in Table 1. To amplify the *ompB* gene, additional new primers targeting the conserved region of the *ompB* gene were used for a semi-nested PCR (sn-PCR) (Table 1). To amplify the *gltA* gene, an additional pair of primers was used: CS151F and CS1259R [25]. Two microliters of specimen DNA was used in a total reaction volume of 25 μl, containing 0.3 μM of each primer (Eurofins MWG Operon, Louisville, KY), 1× Platinum^®^ PCR Supermix, High Fidelity (Invitrogen Corp., Carlsbad, CA) and RNase/DNase free water (GIBCO BRL Life Technologies, Inc., Gaithersburg, MD, USA). One microliter of PCR product was used in the nPCR or snPCR reactions performed in a Veriti Thermocycler (Applied Biosystems, Foster City, CA). For the new primers, the reaction mix was incubated at 94°C for 1 min followed by 40 cycles (35 cycles for nPCR and snPCR) of denaturation at 94°C for 30 seconds, annealing at 54°C (*ompB*) for 30 seconds and elongation at 68°C for 1 min, and final elongation at 72°C for 7 min. All PCR products were visualized using gel red (Biotium, Hayward, CA) on 1% (w/v) agarose gel (Sigma, St Louis, MO, USA). The size of the amplified product was determined by comparison with 1 kb plus molecular marker (Invitrogen). No positive controls were used in the PCR, nPCR, or snPCR procedures to decrease chances of contamination; however, negative controls (molecular biology grade water, GIBCO) were assessed with the samples and they were consistently negative in all runs.

**Table 1:** Oligonucleotide primers.

Gene	Primer	Sequence (5–3)	Reference
17kDa	R17K128F2∞	GGGCGGTATGAAYAAACAAG	(32)
	R17K238R∞	CCTACACCTACTCCVACAAG	(32)
	R17K202TaqP∞	FAM-CCGAATTGAGAACCAAGTAATGC-TAMRA	(32)
*rrs*	16SU17F*	AGAGTTTGATCCTGGCTCAG	(34)
	16SR34F#^	CAGAACGAACGCTATCGGTA	(34)
	16SU1592R*	AGGAGGTRATCCAGCCGCA	(34)
	16SOR1198R#^	TTCCTATAGTTCCCGGCATT	(34)
	16sOR155F^	TCAGTACGGAATAACWTTTAGAAATAA	This study
	16SU547F^	CAGCAGCCGCGGTAATAC	This study
	16sU 833R^	CTACCAGGGTATCTAATCCTGTT	This study
17kDa	R17k31F*#^	GCTCTTGCAGCTTCTATGTTACA	34
	R17k469R*	ACTTGCCATTGTCCGTCAGGTTG	34
	R17k2608R#^	CATTGTCCGTCAGGTTGGCG	34
*ompB*	120-M59F*	CCGCAGGGTTGGTAACTGC	35
	120-1570R*	TCGCCGGTAATTRTAGCACT	34
	120-2788F*^	AAACAATAATCAAGGTACTGT	36
	120-4346R*^	CGAAGAAGTAACGCTGACTT	36
	RompB3521F^	GATAATGCCAATGCAAATTTCAG	36
	RompB4224F^	ACCAAGATTATAAGAAAGGTGATAA	36
	RompB3637R^	GAAACGATTACTTCCGGTTACA	36
	RompB3008R^	CGCCTGTAGTAACAGTTACAC	36
	RompB3503F*#^	ACAACCATTAACGTACAAGATAA	This study
	RompB4293R*	GCACTACCTTGAGCAAAGAA	This study
	RompB4246R#^	CACCTTTCTTATAATCTTGGTGTTTTAT	This study
*gltA*	CS49F*#	ACCTATACTTAAAGCAAGTATYGGT	This study
	CS1273R*	CATAACCAGTGTAAAGCTG	(36)
	CS1234R#	TCTAGGTCTGCTGATTTTTTGTTCA	(36)
*sca4*	RrD749F*	TGGTAGCATTAAAAGCTGATGG	(36)
	RrD928F#^	ATTTATACACTTGCGGTAACAC	(36)
	RrD2685R*#^	TTCAGTAGAAGATTTAGTACCAAAT	(36)
	RrD_Sca4_F3^	CAGGACCTCCTTTGCCTTCAT	This study
	RrD_Sca4_R2^	TGCTTTATCTTGACCGTTCAA	This study
*ompA*	190-70F*	ATGGCGAATATTTCTCCAAAA	(33)
	190-701R*	GTTCCGTTAATGGCAAGCATCT	(33)
	190-602n*	AGTGCAGCATTCGCTCCCCCT	(33)
	190-3588F*#	AACAGTGAATGTAGGAGCAG	(33)
	190-5238R*#	ACTATTAAAGGCTAGGCTATT	(33)

^∞^primers used for qPCR;

*Primers used for PCR amplification;

^#^primers used for nested PCR;

^Primers used for sequencing

PCR products were purified using QIAquick PCR purification kit (QIAGEN). Sequencing reactions were performed utilizing both DNA strands with Big Dye Terminator v3.1 Ready Reaction Cycle Sequencing Kit (Life Technologies, Foster City, CA), according to the manufacturer’s instructions on an ABI 3500 genetic analyzer (Applied Biosystems, Foster City, CA). The *rrs*, *gltA*, 17kDa, *ompB*, and *sca4* sequences were assembled using CodonCode Aligner version 5.0.1 (CodonCode Corporation, Centerville, MA) and exported to Molecular Evolutionary Genetics Analysis (MEGA) version 7 software (CEMI, Tempe, AZ) where they were compared with other historical strains available in GenBank. Phylogenetic analyses were performed using MEGA v7 [37] using Maximum Likelihood (ML) method using the Tamura-Nei model [38] for the individual genes. Multi-locus sequence typing (MLST) phylogeny was performed utilizing concatenated gene fragments of *rrs* (881-bp), *gltA* (993-bp), *ompB* (604-bp), and 17kDa gene (410-bp) of the novel *Rickettsia* genotypes from the mosquitoes in this study and validly published *Rickettsia* species from GenBank. The concatenated alignment tree was also estimated using the ML method and the Tamura-Nei model. Additionally, comparison of the *gltA* and *sca4* sequences from this study and other mosquito molecular isolates was performed to determine their relatedness. Bootstrap analyses were performed with 1,000 replications.

### GenBank accession numbers

The sequences of the new rickettsial molecular isolates A12.2646 from *Mansonia uniformis*, A12.2638 from *Culex pipiens* and A12.3271 from *Aedes esoensis* have been deposited in GenBank with accession numbers KY799063-KY799067, KY799068-KY799071 and KY799072-KY799074, MF590070 for *rrs*, 17 kDa antigen gene, *ompB*, *gltA*, and *sca4*, respectively.

All of the surveillance was done under a United States Forces Korea (USFK) regulation (USFK 2009, Reg. 40–2). We obtained verbal permission from the residents to set a mosquito magnet at two private sites at Daeseongdong and Tongilchon. All the other sites were military installations [Neutral Nations Supervisory Commission (NNSC) camp] or training sites (South Gate to the DMZ which is part of the Joint Security Area and Warrior Base).

## Results

Metagenome sequencing were conducted for a total of 240 mosquito pools collected at four locations (NNSC camp, Daeseongdong village, beef farm at Tongilchon, and WBTA near/in the DMZ in the ROK. Pools included a total of 2,248 mosquitoes belonging to five genera and seven species, including: *Aedes esoensis* (n = 456), *Armigeres subalbatus* (n = 94), *Culex bitaeniorhynchus* (n = 1007), *Culex pipiens* (n = 627), *Mansonia uniformis* (n = 19), *Ochlerotatus koreicus* (n = 40), and *Ochlerotatus nipponicus* (n = 5).

Analyses of metagenome sequencing data identified rickettsial ribosomal RNA genes, suggesting the presence of rickettsiae in eight pools (Table 2). The eight positive pools and seven additional *M*. *uniformis* pools (negative for rickettsial RNA in NGS) were subjected to further molecular analyses. To confirm the presence of *Rickettsia* nucleic acid, a genus-specific qPCR (Rick17b) assay was used that showed that 7/8 (87.5%) rickettsial rRNA positive mosquito DNA pools were also positive for *Rickettsia* DNA, while, all seven pools of rickettsial rRNA negative *M*. *uniformis* were confirmed negative for rickettsial DNA. The seven *Rickettsia*-positive mosquito samples included: 3 pools of *Cx*. *pipiens*, 3 pools of *Ae*. *esoensis* and 1 pool of *Mn*. *uniformis*. One pool of *Cx*. *bitaeniorhynchus* that was positive for rickettsial RNA by NGS was negative by the Rick17b assay.

**Table 2:** Summary of qPCR and sequencing results for mosquito samples obtained from the Republic of Korea.

							Sequencing					
Sample Name	Location	Mosquito Species	Mosquitoes/ pool	Date collected	NGS	Rick17b qPCR	*rrs*	17kDa	*gltA*	*ompB*	*ompA*	*sca4*
A12.2552	Daeseongdong	*Cx*. *bitaeniorhynchus*	30	20-Aug-12	Pos	Neg	-	-	-	-	-	-
A12.2638	Daeseongdong	*Cx*. *pipiens*	30	27-Aug-12	Pos	Pos	+	+	+	+	-	-
A12.2639	Daeseongdong	*Cx*. *pipiens*	19	27-Aug-12	Pos	Pos	+	+	+	-	-	-
A12.2646	Daeseongdong	*Mn*. *uniformis*	2	27-Aug-12	Pos	Pos	+	+	+	+	-	+
A12.2784	Warrior base	*Cx*. *pipiens*	17	3-Sep-12	Pos	Pos	+	+	+	+	-	-
A12.2856	Daeseongdong	*Ae*. *esoensis*	30	6-Sep-12	Pos	Pos	+	+	+	+	-	-
A12.3106	NNSC	*Ae*. *esoensis*	20	17-Sep-12	Pos	Pos	+	+	+	+	-	-
A12.3271	NNSC	*Ae*. *esoensis*	13	24-Sep-12	Pos	Pos	+	+	+	+	-	-

NGS- Next Generation Sequencing

Amplicons were generated for one or more of the rickettsial gene targets; *rrs* (7/7), *gltA* (7/7), 17kDa (7/7), *ompB* (6/7) and *sca4* (1/7) (Table 1). Attempts to amplify the *ompA* fragments were unsuccessful for all samples. No culicine mosquitoes collected from Tongilchon assayed by NGS were positive for rickettsiae.

The sequences of the *rrs* from the seven Rick17b positive mosquito pools were compared to those available in the GenBank. The sequence from one mosquito pool of *Cx*. *pipiens* (A12.2638) shared 100% sequence identity with *Rickettsia sp*. WHANSA-97 from *An*. *sinensis* from China (KU586119) and 99.9% with *Rickettsia bellii* RML369-C. The sequences of the other 6 pools shared 99.3% sequence identity with *R*. *bellii* RML369-C. The *rrs* sequences from all the other samples exhibited ≤98.2 sequence homology with rickettsiae identified in mosquitoes *Cx*. *quinquefasciatus*, *An*. *sinensis* and *Cx*. *tritaeniorhynchus* from China [25] (Table 3).

**Table 3:** Percent sequence identity of the mosquito genotypes to *Rickettsia* species with validly published names and other mosquito rickettsial strains provided in the GenBank.

Gene	Mosquito pool	Mosquito species	Sequence length	Percent identity with the closest *Rickettsia* sp. (number of matches/sequence length)	Accession number	Fournier et al. (41) Cutoff values
*rrs*	A12.2638	*Culex pipiens*	1091	99.9% (1087/1088) *R*. *bellii*	CP000087	≥99.8%
				99.9% (806/807) *R*. *bellii* WHANSA-97_*An*. *sinensis*	KU586119	
				98.1% (1006/1026) *R*. *monacensis*_*Cx*. *quinquefasciatus*	KU586120	
				97.5% (384/394) *Candidatus* R. sp. *An*. *sinensis*	KU586118	
				95.5% (362/379) *Candidatus* R. sp. Cx. *tritaeniorhynchus*	KU586123	
	A12.2646	*Mansonia uniformis*	1096	99.3% (1083/1091) *R*. *bellii*	CP000087	
	A12.2639	*Culex pipiens*	1090	99.3% (1082/1090) *R*. *bellii*	CP000087	
	A12.2784	*Culex pipiens*	1090	99.3% (1082/1090) *R*. *bellii*	CP000087	
	A12.2856	*Aedes esoensis*	1091	99.3% (1083/1091) *R*. *bellii*	CP000087	
	A12.3106	*Aedes esoensis*	1090	99.3% (1082/1090) *R*. *bellii*	CP000087	
	A12.3271	*Aedes esoensis*	1419	99.3% (1409/1419) *R*. *bellii*	CP000087	
				99.3% (1093/1101) *R*. *bellii* WHANSA-97_*An*. *sinensis*	KU586119	
				98.2% (1291/1314) *R*. *monacensis*_*Cx*. *quinquefasciatus*	KU586120	
				97.5% (668/685) *Candidatus* R. sp. *An sinensis*	KU586118	
				96.4% (646/670) *Candidatus* R. sp. Cx. *tritaeniorhynchus*	KU586123	
17kDa	A12.2638	*Culex pipiens*	410	89.3% (367/411) *R*. *helvetica*	LN794217	NA
	A12.2646	*Mansonia uniformis*		98.0% (402/410) *R*. *monacensis*	LN794217	
				100.0% (394/394) *Rickettsia* sp. Tx140	EF689738	
	A12.2639	*Culex pipiens*		89.3% (367/411) *R*. *helvetica*	LN794217	
	A12.2784	*Culex pipiens*		89.3% (367/411) *R*. *helvetica*	LN794217	
	A12.2856	*Aedes esoensis*		89.3% (367/411) *R*. *helvetica*	LN794217	
	A12.3106	*Aedes esoensis*		89.3% (367/411) *R*. *helvetica*	LN794217	
	A12.3271	*Aedes esoensis*		89.3% (367/411) *R*. *helvetica*	LN794217	
*ompB*	A12.2638	*Culex pipiens*	640	85.0% (192/226) *R*. *hoogstraalii*	EF629536	≥99.2%
	A12.2646	*Mansonia uniformis*	690	94.1% (649/690) *R*. *hoogstraalii*	EF629536	
				91.1% (626/687) *Rickettsia* sp. *Anopheles gambiae*	JN620080	
				90.7% (514/567) *Rickettsia* sp. *Culex pipiens*	KU761260	
	A12.2639	*Culex pipiens*		-	-	
	A12.2784	*Culex pipiens*	646	85.3% (198/232) *R*. *hoogstraalii*	EF629536	
	A12.2856	*Aedes esoensis*	646	85.3% (198/232) *R*. *hoogstraalii*	EF629536	
	A12.3106	*Aedes esoensis*	646	85.3% (198/232) *R*. *hoogstraalii*	EF629536	
	A12.3271	*Aedes esoensis*	649	85.5% (201/235) *R*. *hoogstraalii*	EF629536	
*gltA*	A12.2638	*Culex pipiens*	1004	97.8% (982/1004) *R*. *bellii*	CP000087	≥99.9%
				100% 1004/1004) *R*. *bellii* WHANSA-97_An. sinensis	KU586331	
	A12.2646	*Mansonia uniformis*	995	96.5% (960/995) *R*. *asembonensis*		
				96.7% (695/719) *Rickettsia* sp. *Anopheles melas*	JQ354961	
				96.5% (920/953) *Rickettsia* sp. *Anopheles gambiae*	JN620082	
				96.1% (366/381) *Rickettsia* sp. *Culex pipiens*	KU761259	
				94.7% (942/995) *R*. *monacensis*_*Cx*. *quinquefasciatus*	KU586332	
	A12.2639	*Culex pipiens*	1000*	85.4% (854/1000) *Rickettsia felis*	CP000053	
	A12.2784	*Culex pipiens*		84.9% (849/1000) *R*. *bellii*	CP000087	
	A12.2856	*Aedes esoensis*		96.5% (920/953) *Rickettsia* sp. *Anopheles gambiae*	JN620082	
	A12.3106	*Aedes esoensis*				
	A12.3271	*Aedes esoensis*				
*sca4*	A12.2638	*Culex pipiens*		-	-	≥99.3%
	A12.2646	*Mansonia uniformis*	1681	92.3% (1559/1689) *R*. *felis*	CP000053	
				98.5% (326/331) *Rickettsia* sp. *Culex pipiens*	KU761261	
				81.8% (516/631) *Rickettsia* sp. *Anopheles gambiae*	JN620081	
	A12.2639	*Culex pipiens*		-	-	
	A12.2784	*Culex pipiens*		-	-	
	A12.2856	*Aedes esoensis*		-	-	
	A12.3106	*Aedes esoensis*		-	-	
	A12.3271	*Aedes esoensis*		-	-	

NA: not applicable—Not amplified

*-*gltA* sequences from the five pools were identical represented (A12.3271 pool)

Seven 410-bp sequences of the 17kDa antigen gene were generated from all seven positive mosquito pools (Table 3). The sequence from pool A12.2646 was only 98.0% identical to the closest *Rickettsia* with a validly published name, *R*. *monacensis* IrR/Munich. A 394-bp fragment of this gene sequence was 100% identical to an uncultured *Rickettsia* sp. Tx140 (EF689738) from an *Ixodes scapularis* tick from Texas, USA. The other six sequences (A12.2638, A12.2639, A12.2784, A12.2856, A12.3106 and A12.3271) were 100% identical to each other and were most similar to, yet very disparate from *R*. *helvetica* (89.3%) and *R*. *monacensis* (88.3%).

Attempts to amplify the *ompB* gene using previously described primers [34, 35], were unsuccessful. However, successful amplification of the pool A12.2646 using previously described primers [36] was accomplished. While using the new *ompB* primers, snPCR produced amplicons of the appropriate size from 6 mosquito pools (Table 3). A 690-bp *ompB* sequence from the *Mn*. *uniformis* pool, A12.2646 was identical to the sequence produced by 120-2788F/120-4346 primer pair, and was distantly related to *R*. *hoogstraalii* (94.1%). An additional 687-bp and 567-bp sequence of the *ompB* gene shared only 91.1% and 90.7% nucleotide similarity with the uncultured *Rickettsia* sp. from *An*. *gambiae* and *Rickettsia* endosymbiont of *Cx*. *pipiens*, respectively. The *ompB* sequences from five other mosquito pools (A12.2638, A12.2784, A12.2856, A12.3106 and A12.3271) were quite different from any other known *Rickettsia* sp. Only 34% of the *ompB* sequence aligned to *R*. *hoogstraalii* at ≤85.5% sequence homology.

First, attempts to amplify the *gltA* gene fragment using primer CS49F and previously described primers [36] were unsuccessful. Alternatively, primers previously described [25] were used and a 995-1004-bp *gltA* gene sequences were amplified from all the seven mosquito pools. Pool A12.2638 *gltA* sequence was 100% similar to *Rickettsia sp*. WHANSA-97 from *An*. *sinensis* but exhibited only 97.8% similarity with *R*. *bellii* RML369-C. Pool A12.2646 *gltA* sequence was most similar to that of *R*. *asembonensis* (96.5%) isolated from cat fleas. The sequences from mosquito samples A12.2639, A12.2784, A12.2856, A12.3106 and A12.3271 were identical and shared only 84.9% sequence similarity with *R*. *bellii*-the closest *Rickettsia* species. However, the *gltA* sequences shared ≤96.7% nucleotide identity in the respective aligned regions with uncultured *Rickettsia* spp. from *An*. *melas*, *An*. *gambiae*, and *Rickettsia* endosymbiont of *Cx*. *pipiens* (Table 3). The *gltA* sequences indicate that the rickettsiae from the pooled mosquitoes are genetically unique.

A 1,681-bp sequence from the *sca4* gene was produced in 1/7 (14.3%) mosquito pools (A12.2646). Based on this gene, the sequence exhibited the highest similarities to *R*. *felis* URRWXCal2 (92.3%), and shared 98.5% and 81.8% nucleotide identity with the *Rickettsia* endosymbiont of *Cx*. *pipiens* and uncultured *Rickettsia* sp. from *An*. *gambiae*, respectively (Table 3).

**Fig 2:**
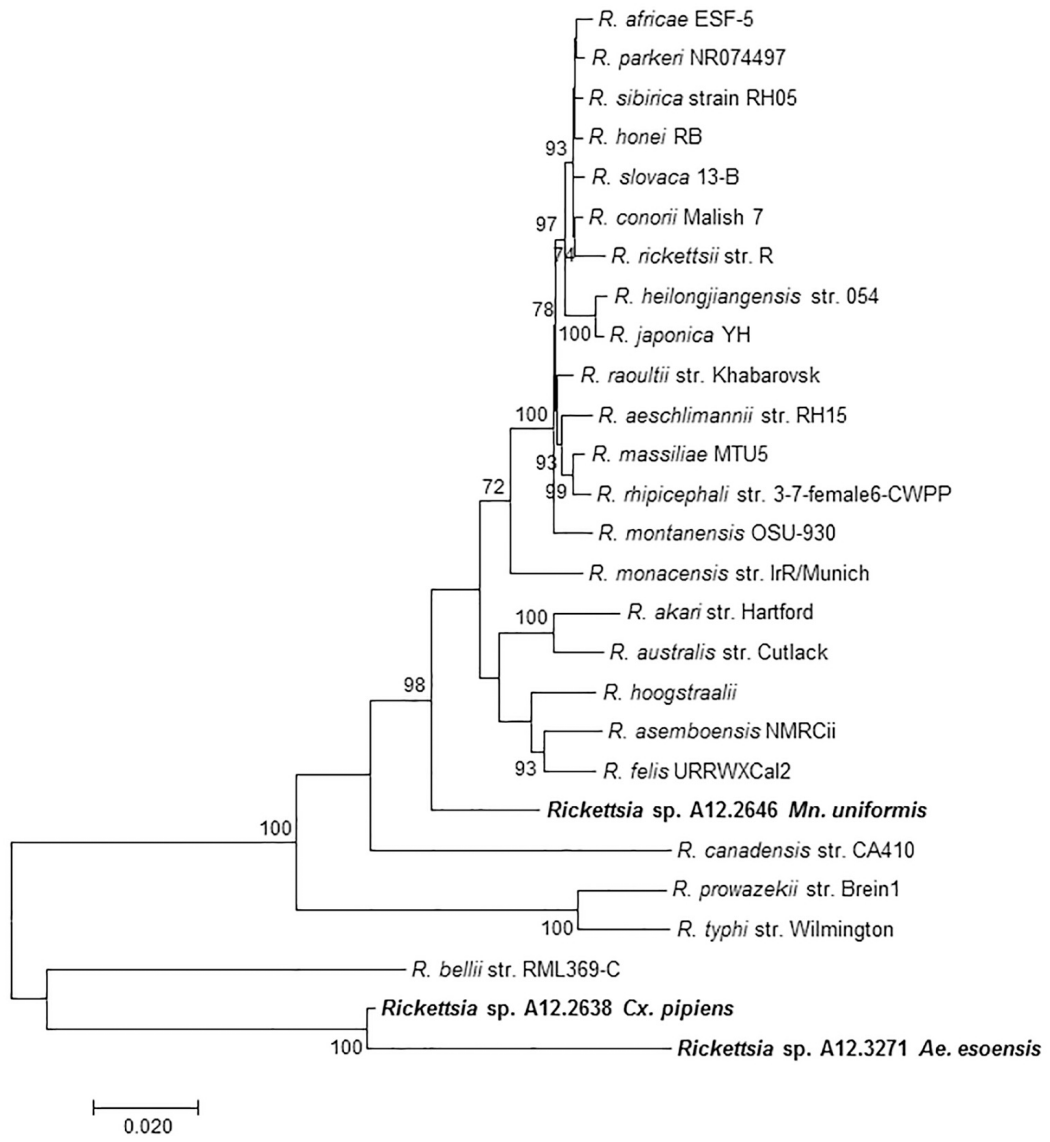
MLST dendrogram consisting of concatenated gene fragments of: *rrs* (881), *gltA* (993), *ompB* (604) and 17kDa gene (410) showing the phylogenetic position of *Rickettsia* sp. A12.2646 from *Mansonia uniformis*, *Rickettsia* sp. A12.2638 from *Cx*. *pipiens* and A12.3271 from *Ae*. *esoensis* among *Rickettsia* species in the GenBank. The evolutionary history was inferred by the maximum likelihood method.

**Fig 3:**
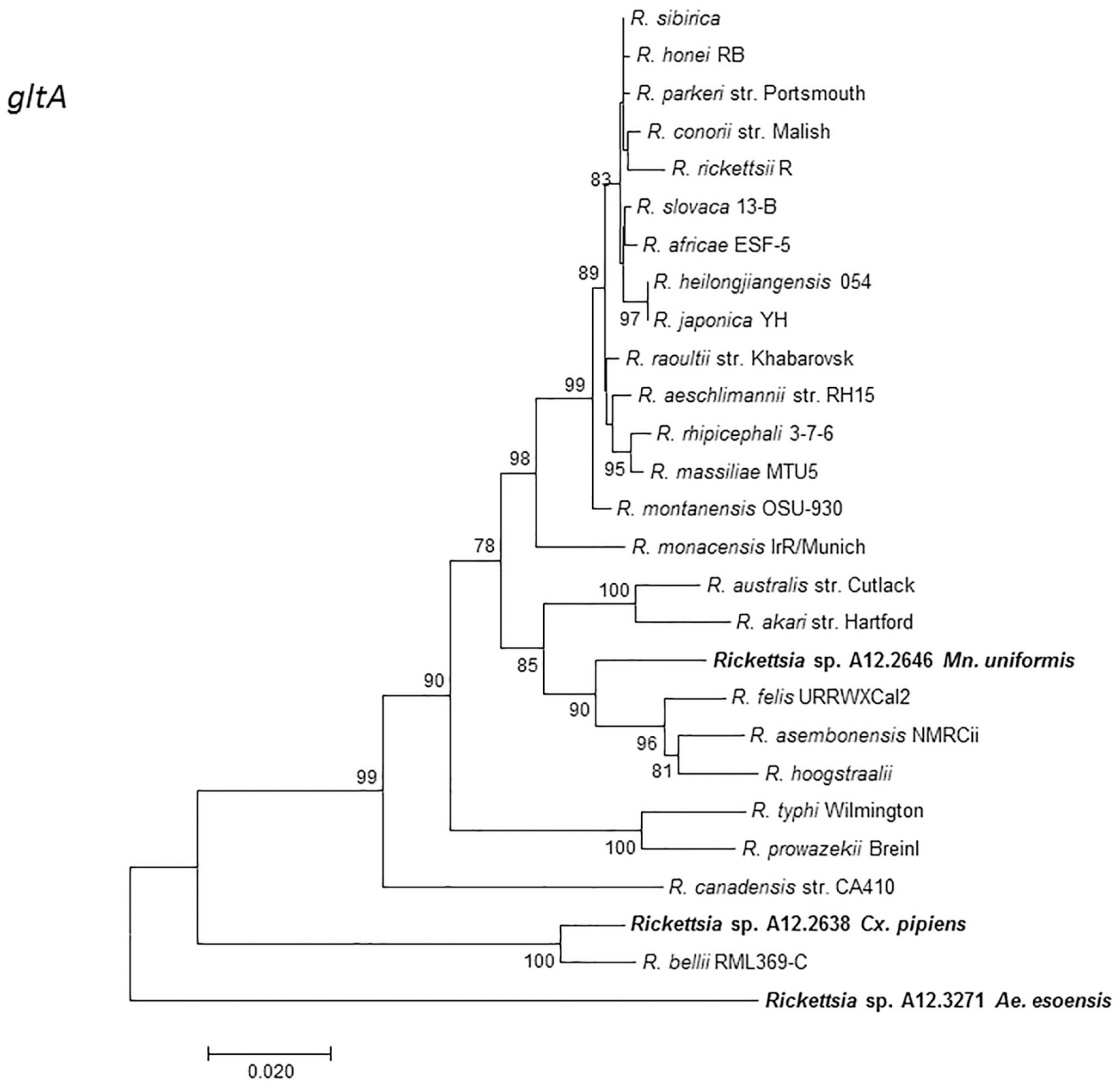
A dendrogram showing the phylogenetic position of *Rickettsia* sp. A12.2646 from *Mn*. *uniformis*, *Rickettsia*. sp. A12.2638 from *Cx*. *pipiens* and A12.3271 from *Ae*. *esoensis* from the Republic of Korea in relation to other *Rickettsia* species with validly published names for the *gltA* gene (993-bp). The evolutionary history was inferred by the maximum likelihood method.

**Fig 4:**
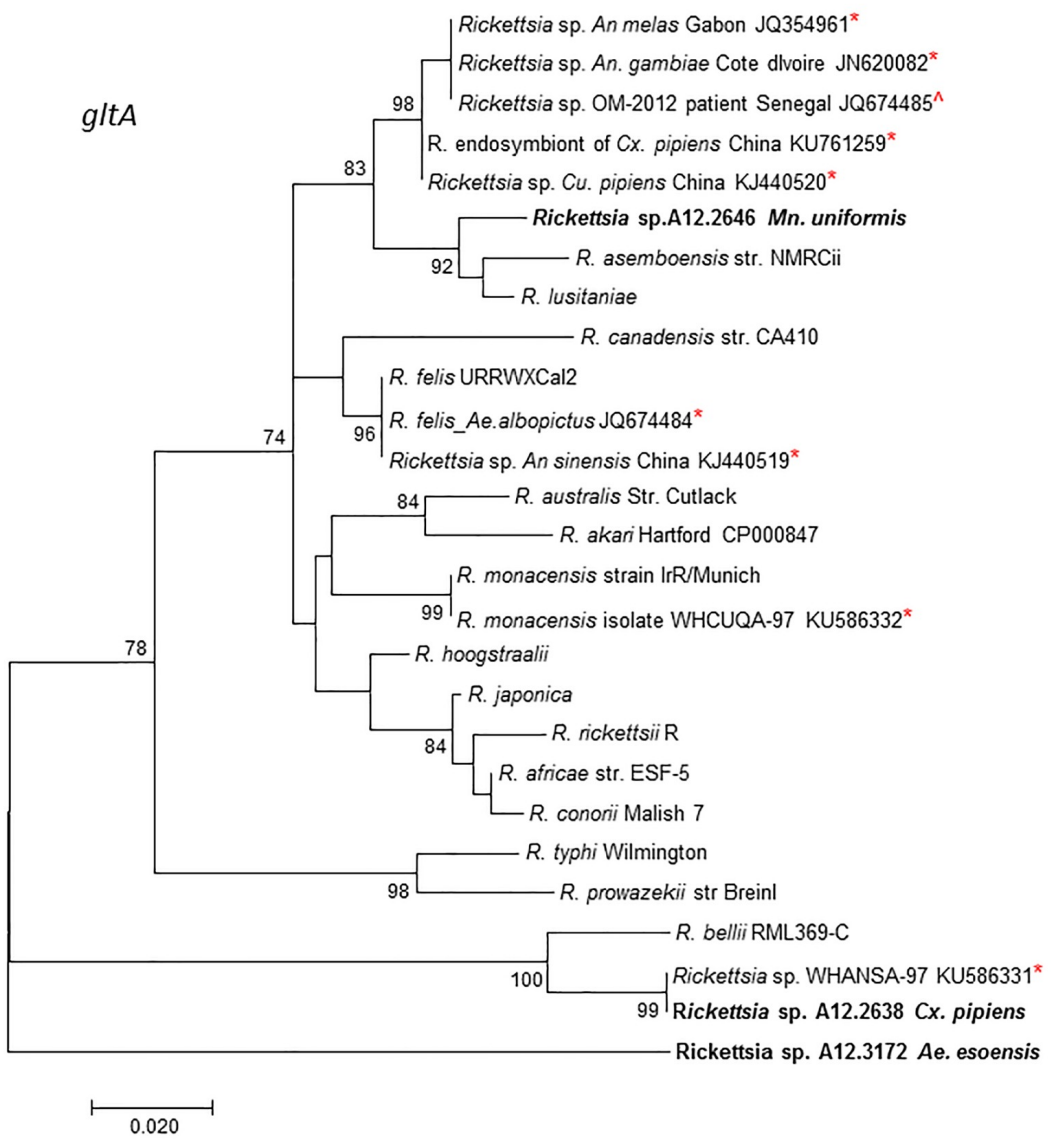
A dendrogram based on the *gltA* gene (345-bp) showing the phylogenetic position of *Rickettsia* sp. A12.2646 from *Mn*. *uniformis*, *Rickettsia* sp. A12.2638 from *Cx*. *pipiens* and A12.3271 from *Ae*. *esoensis* from Republic of Korea in relation to other rickettsial genotypes from mosquitoes and other historical strains provided in GenBank. The evolutionary history was inferred using maximum likelihood method. *Represent other *Rickettsia* sp. from mosquitoes and ^from a patient’s blood.

MLST analysis using concatenated fragments of *rrs*, *gltA*, 17kDa, and *ompB* genes showed that the *Rickettsia* sp. A12.2646 from *Mn*. *uniformis* did not cluster with any *Rickettsia* species but branched separately between the SFGR group and *R*. *canadensis*. *Rickettsia* sp. A12.2638 from *Cx*. *pipiens* and *Rickettsia* sp. A12.3271 from *Ae*. *esoensis* branched separately in a clade basal to *R*. *bellii* (Fig 2). Phylogenetic analysis based on the *gltA* gene sequences showed that *Rickettsia* sp. A12.2638 from *Cx*. *pipiens* clustered together with *R*. *bellii* RML369-C while *Rickettsia* sp. A12.3271 from *Ae*. *esoensis* was placed in a separate branch basal to the *R*. *bellii*. *Rickettsia* sp. A12.2646 from *Mn*. *uniformis* branched separately from the other members of SFGR (Fig 3). Incorporating other mosquito rickettsial genotypes detected in Africa and China, *Rickettsia* sp. A12.2638 from *Cx*. *pipiens* clustered together with *R*. *bellii* RML369-C and *Rickettsia sp*. WHANSA-97 detected in *An*. *sinensis* from China, while *R*ickettsia sp. A12.2646 from *Mn*. *uniformis* was placed in a clade together with other members of SFGR, but in a different branch away from other *Rickettsia* species from mosquitoes (Fig 4). Similar divergence was observed with the *sca4* gene sequence from *Rickettsia* sp. A12.2646 (S1 Fig). The sequences from mosquito samples A12.2639, A12.2784, A12.2856, A12.3106 and A12.3271 were identical for *rrs*, *ompB* and 17kDa, and for the purpose of illustration, they are represented by A12.3271 from *Ae*. *esoensis*. The *ompB* sequence from A12.2639 was not amplifiable.

## Discussion

Recent reports suggesting that mosquitoes may play a role in the epidemiology of *R*. *felis* rickettsiosis [19] led us to conduct this study to determine whether *R*. *felis* and/or other rickettsiae could be detected in mosquitoes collected near/in the DMZ using NGS metagenome sequence-based pathogen discovery studies [30, 39]. Utilizing NGS, qPCR and MLST, three novel *Rickettsia* agents were detected in three different mosquito species: *Cx*. *pipiens*, *Mn*. *uniformis* and *Ae*. *esoensis*.

NGS has been used to unearth novel microbes including rickettsiae in ticks in western Europe [40, 41]. Our study used NGS for the detection of rickettsiae in mosquitoes, which resulted in the discovery of three potentially new *Rickettsia* genotypes in mosquito hosts. Though the random unbiased manner of metagenome NGS led to the production of a limited amount of species specific sequences that were insufficient to discriminate rickettsiae to species level with confidence, it is a great means of screening for rickettsiae as shown in this report. To overcome this limitation, mosquito pools positive for rickettsiae by NGS were subsequently evaluated using a *Rickettsia*-genus specific qPCR assay and the species determined by MLST. This application demonstrated a good concordance between NGS and the genus-specific qPCR assay, with 7/8 NGS positive samples being confirmed positive by Rick17b qPCR assay. *Rickettsia* DNA was not detected by the Rick17b assay in the mosquito DNA preparation from *Cx*. *bitaeniorhynchus* that was positive by NGS. The inability to amplify rickettsial DNA from *Cx*. *bitaeniorhynchus* preparation using the *Rickettsia* genus-specific qPCR (Rick17b) may be due to its presence in low copy numbers that were below the detection limit of the Rick17b assay or because the gene may be too divergent for amplification using the Rick17b primers.

Previous studies have documented the presence of rickettsiae in mosquitoes using species-specific qPCR assays and/or by PCR and sequencing of one or more of the following gene fragments; *rrs*, *gltA*, *ompA*, *ompB*, *sca4*, and *groEL*. A high rate of infection was reported in a recent study, where 21% of 53 *Cx*. *pipiens* mosquitoes assessed were infected with a novel *Rickettsia* [18]. Additionally, extensive diversity of Rickettsiales in multiple mosquito species was reported by Guo et al. [25]. In this study, there were three novel *Rickettsia* genotypes in *Mn*. *uniformis*, *Cx*. *pipiens*, and *Ae*. *esoensis* identified, which corroborate previous reports of the occurrence of *Rickettsia felis* [21, 26, 27] and other novel genotypes [18, 23, 25] in numerous mosquito species. Although there is no proof that vertical transmission of rickettsiae in these blood feeders occur, Guo et al. [25] demonstrated rickettsiae in each life stage (eggs, larvae, pupae, and adults) of laboratory-cultivated *Armigeres subalbatus* and *Cx*. *tritaeniorhynchus* mosquitoes. This observation suggests transovarial and transstadial transmission of rickettsiae in mosquitoes, and is corroborated by a previous study that detected *R*. *felis* in a male *An*. *arabiensis* mosquito [21]. However, in a separate laboratory study, when mosquitoes were fed by membrane feeding on blood infected with *R*. *felis*, the mosquitoes became infected but the bacteria were not transmitted to the F_1_ progeny [19]. This is the first report of detection of rickettsiae in *Ae*. *esoensis* and in mosquitoes from ROK. It was not determined if the rickettsiae are endosymbionts with transovarial transmission or if the mosquitoes became infected with rickettsiae through an alternative method. This report adds to the list of mosquito species that harbor *Rickettsia* species and shows that they may be more prevalent in mosquitoes than previously thought.

To identify the mosquito-borne rickettsiae we utilized three conserved gene fragments (*rrs*, 17 kDa antigen gene, and *gltA*) and three variable gene fragments (*ompA*, *ompB*, and *sca4*). Successful amplification and sequencing was achieved for all seven mosquito preparations for the *rrs* and 17kDa genes, but was variably successful using other gene fragments (*gltA*, *ompB*, and *sca4*) or unsuccessful (*ompA*). The much conserved *rrs* gene fragment sequences from *Rickettsia*-positive mosquitos (*Cx*. *pipiens*, *Mn*. *uniformis* and *Ae*. *esoensis*) were closest to that of *Rickettsia* (99.3–99.9%) in GenBank and confirmed the presence of rickettsiae in mosquitoes. Attempts to amplify various fragments of the variable *ompB* gene were somewhat successful. Similarly, a previous study encountered [23] comparable problems with mosquito preparations that were positive for *Rickettsia* spp. by qPCR, but could not be amplified by various primers targeting the *ompB* gene. Amplification was achieved herein using the primer pair 120–2788 and 120–4346, for only one mosquito pool (A12.2646 from *Mn*. *uniformis*) and in 6/7 mosquito pools using a new set of primers. The *ompB* sequence from five mosquito DNA preps was very divergent with >65% of the *ompB* sequence being unable to identify any match in the GenBank. This indicates that the rickettsiae from these pooled mosquito samples are genetically unique. We were most likely unable to differentiate the sequences from these five mosquito pools because the amplified region was in a highly conserved region from which our primers were located (4293–3503) as shown in other studies [42].

BLASTN searches for the *rrs*, *gltA*, *ompB*, and *sca4* genes among mosquito-borne rickettsiae identified numerous sequences from mosquito species from China and Africa. Even though these rickettsiae came from similar hosts, none of the uncultured *Rickettsia* spp. identified in these mosquitoes including *Candidatus* Rickettsia sp. *An*. *sinensis*, *Candidatus* Rickettsia sp. *Culex tritaeniorhyncus* [25], the two uncultured *Rickettsia* spp. detected in *An*. *gambiae* and *An*. *melas* mosquitoes from Gabon [23] and a *R*. endosymbiont found in *Cx*. *pipiens* from China [18] shared perfect identity with the ones detected in the present study. However, the highly conserved *rrs* and *gltA* sequences from A12.2638 shared high nucleotide similarities (99.9% and 100%, respectively) with a *Rickettsia* sp. WHANSA-97 detected in *An*. *sinensis* from China. While it is likely that these two are the same, or very closely related, BLASTN searches for the *gltA* gene sequences revealed 97.8% or less homology with any of the *R*. *bellii* with validly published names. Additionally, the *ompB* sequence from A12.2638 could not identify any similarities with *R*. *bellii*. Due to the significant differences in the *gltA* and *ompB* sequences, we do not believe that this is *R*. *bellii*, but maybe related.

A previous study detected *R*. *felis* in *Mn*. *uniformis* from Senegal [21]. This is in contrast to the finding from this study where a different genotype was identified in a pool (A12.2646) of two *Mn*. *uniformis* mosquitoes. This suggests that one species of mosquito is capable of hosting different rickettsial species. The rickettsiae identified from A12.2646 clustered with *R*. *felis*-like organisms and other uncultured *Rickettsia* sp. from *An*. *gambiae* and *An*. *melas* [18, 23], and shared only ≤ 96.7% *gltA* nucleotide sequence homologies with the latter. Interestingly, the *gltA* sequences from the uncultured *Rickettsia* spp. detected in *An*. *gambiae* and *An*. *melas* mosquitoes from Gabon were closely related (99.7%) to a *Rickettsia* sp. detected in blood from a febrile patient from Senegal [JQ674485] [23]. This close relationship might indicate that some of these uncultured *Rickettsia* species found in mosquitoes may be potential human pathogens. Further work to establish the transmission dynamics of mosquito-borne rickettsiae and their potential to infect mammalian hosts is needed.

To strengthen the characterization of the mosquito-borne rickettsiae within the genus we concatenated *rrs*, *gltA*, 17 kDa gene, and *ompB* gene fragments. Unfortunately, we did not include the other genes because there was only one sequence obtained using *sca4*, and none from *ompA*. Based on the phylogenetic relationship inferred by concatenation of the *rrs*, *gltA*, 17kDa, and the *ompB* gene fragments, *Rickettsia* sp. A12.2646, *Rickettsia* sp. A12.2638, and *Rickettsia* sp. A12.3271 branched separately to form distinct lineages. The novel mosquito species, *Rickettsia* sp. A12.2638 and *Rickettsia* sp. A12.3271 were placed in a branch basal to the *R*. *bellii* clade, which supports the presence of additional rickettsial lineages [1], other than the widely accepted groups of the SFGR, TG, transitional group *Rickettsia* (TGR), and an ancestral group *Rickettsia* (AGR).

The nucleotide sequence similarities of the rickettsiae from mosquitoes collected from ROK were lower than the cut-off values proposed for *Rickettsia* species definition [43] and suggest them as tentative new *Rickettsia* species. Further studies are needed to shed light on the evolutionary relationship of these mosquito-borne rickettsiae to previously described rickettsiae, and their seasonal, geographic and species diversity, prevalence, biology, and pathogenic potential.

## Supporting information

S1 Fig A dendrogram based on the sca4 gene (329-bp) showing the phylogenetic position of Rickettsia sp. A12.2646 from *Mn*. *uniformis* from Republic of Korea in relation to other rickettsial genotypes from mosquitoes and other historical strains provided in GenBank. The evolutionary history was inferred using maximum likelihood method. *Represent other Rickettsia sp. from mosquitoes.
(TIF)
