# Supplementary figures and images for: Molecular characterization of novel mosquito-borne Rickettsia spp. from mosquitoes collected at the Demilitarized Zone of the Republic of Korea
Source: PLoS One. 2017 Nov 20;12(11):e0188327. doi: 10.1371/journal.pone.0188327 (PMC5695765; doi:10.1371/journal.pone.0188327)

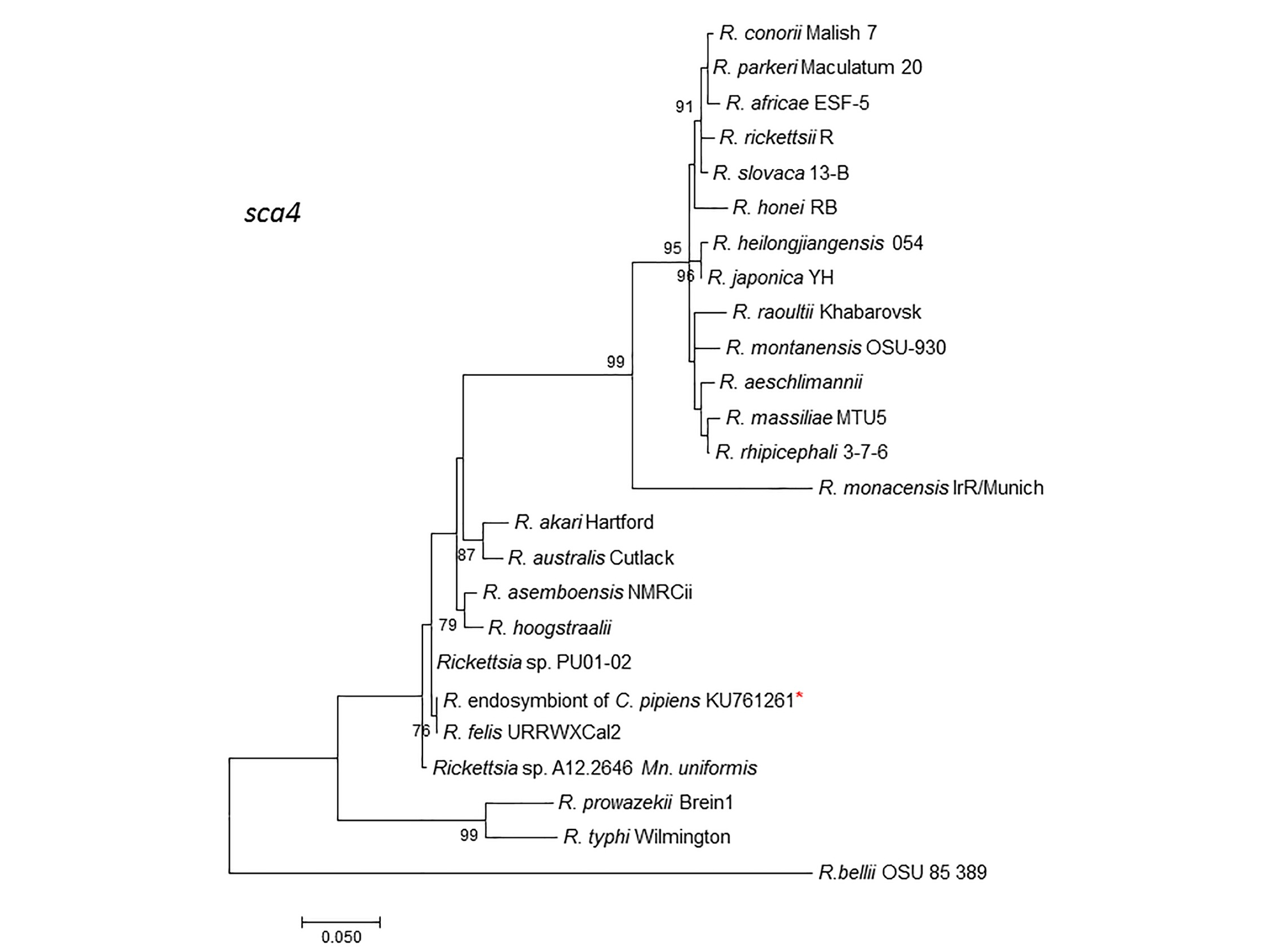

Supplement: S1 Fig — The evolutionary history was inferred using maximum likelihood method. *Represent other Rickettsia sp. from mosquitoes. (TIF) [file pone.0188327.s001.tif]
